# Rapid Screening of Anticoagulation Compounds for Biological Target-Associated Adverse Effects Using a Deep-Learning Framework in the Management of Atrial Fibrillation

**DOI:** 10.3390/bioengineering12090972

**Published:** 2025-09-12

**Authors:** Tim Dong, Rhys Llewellyn, Melanie Hezzell, Gianni D. Angelini

**Affiliations:** 1 Bristol Heart Institute, Translational Health Sciences, University of Bristol, Bristol BS2 8HW, UK; 2 Pharmacy Department, Liverpool Heart and Chest Hospital, Thomas Dr., Liverpool L14 3PE, UK; 3 Bristol Veterinary School, University of Bristol, Langford House, Langford, Bristol BS40 5DU, UK

**Keywords:** deep learning, clinical trials, adverse-effects screening, drug discovery, proteomics, thrombotic disease prevention, atrial fibrillation, machine learning

## Abstract

**Background:** Deep learning methods may be useful for drug compound interaction and discovery analysis. However, there has been limited research on their use for screening biologically related adverse effects. **Objectives:** This study aims to pre-emptively screen for likely drug use persistence or success in clinical trials. **Methods:** This shall be achieved through the extension, application, and evaluation of a deep learning-based framework. Specifically, it shall be considered in the discovery of novel candidates and mechanisms underlying AF management-related adverse effects. The targets were linked to their adverse effects specified in two previous studies, their corresponding protein sequences, and the organs affected. **Results:** The new model showed good performance when compared to existing approaches in the Side Effect Resource (SIDER) and Food and Drug Administration Adverse Event Reporting System (FAERS) external validation datasets. A precision of 0.879 was obtained for enoxaparin, along with a recall of 0.746 for rivaroxaban. Stronger bleeding-related adverse effects were found for edoxaban compared with apixaban and enoxaparin. The binding and safety profiles of sequoiaflavone were very similar to those of rivaroxaban. **Conclusions:** This study presents a framework that could be used to pre-emptively screen for adverse effects. In doing so, it considers the biological basis for guiding optimal drug selection.

## 1. Introduction

Drug development is a high-risk task, which mandates the evaluation of the drug in vitro and in vivo following the design and optimisation of these compounds. This process is time-consuming, typically taking at least 10 to 15 years to complete. Unfortunately, the process is akin to a “shot-in-the dark” that frequently ends up in failure. Technologies are currently being actively researched to facilitate a reduction in the time required for successful drug trial completion. Adverse effects are important endpoints in clinical trials and are one of the major reasons for drug trial failures if detected at higher rates. Asundexian is an example of one such anticoagulant drug that has not progressed beyond phase III trials [1]. Initially, asundexian was associated with good efficacy in terms of fewer bleeding events [2]. However, later on, it demonstrated high rates of stroke and systemic embolism (adverse events) compared with apixaban. Such failed clinical trials highlight the need for advanced technologies to pre-emptively filter and increase the success of drugs put forward for clinical trials.

Currently, there is an under-reporting of side effects in research studies and clinical trials due to factors such as lack of knowledge, lethargy, and complacency [3]. The number or percentage of patients experiencing moderate and serious adverse effects is reported in clinical trials. However, the specific details of the adverse effects are often not provided or discussed. This is counterproductive to the comprehensive assessment of the risk vs. benefits for drug development and clinical decision making. That is, such point-wise quantitative figures do not provide an understanding of the relative severity or impact of individual adverse effects. This publication bias may result partly from the desire of researchers, clinical trials teams, and pharmaceutical companies not to dissuade hospital patients and the public (users of over-the-counter drugs) from using such evaluated drugs.

In actively trialled phase III drugs such as the factor XI-inhibiting drug abelacimab, details of the serious adverse events were provided. However, details of the many moderate adverse events were not provided. Given that 15% of patients experienced greater than 1 moderate adverse event, such information would be important [4]. Similarly, in a comparative trial of abelacimab against rivaroxaban, 358/427 (83%) of patients in this large international clinical trial of AF patients experienced moderate adverse effects of some kind [5]. Further, 157/427 (37%) of patients experienced serious adverse effects [5]. Again, no details of which types of adverse effects were reported. In another study of abelacimab, nine mild adverse effects were considered to be potentially drug-related, but only the most common side effect (headache, 11%) and the side effects unrelated to the drug treatment were reported [5].

Further details of drug adverse effects are generally only available through specific online websites (such as the National Health Service (NHS), British National Formulary (BNF), drug and patient leaflets, etc.). These lists often remain incomplete and are based on yellow card reporting after the drug has been used extensively. For detailed assessment of drug adverse effects, pharmacists typically refer to the clinical summary of the product characteristics section of the electronic medicines compendium (EMC) database under the Healthcare Professionals (SmPC) Section 4.8, Undesirable effects [6]. This may provide more details than the above-mentioned sources, for which simplicity and interpretability are more important.

The majority of machine or deep learning models, after development, are either thrown away or remain unused following publications. This study aims to break away from this trend by extending, applying, and evaluating the deep cross-attention-based modelling framework developed in our previous study [7]. It shall pre-emptively screen for likely drug use persistence or success in clinical trials. The objectives include (i) synthesising a compound structure-based dataset combined with targets and adverse effects from two previous studies; (ii) incorporation of text normalisation modelling component into the existing modelling framework; (iii) assessing the interaction of anticoagulants to protein targets related to bleeding adverse effects; (iv) assessment of overall adverse effects interaction of anticoagulants across organ types.

## 2. Methods

### 2.1. Dataset and Materials

The primary training dataset is the MINER drug–target interaction (DTI) dataset from the BIOSNAP collection (Table 1A) [7]. It includes 13,741 DTI pairs from DrugBank, 4510 drug compounds, and 2181 protein targets. The Database of Useful Decoys (DUD-E) was used to fine-tune the model using contrastive learning. The DUD-E dataset analysed consisted of 57 protein targets and active compounds that are known to interact with these targets, as well as decoys that are known not to bind the targets but have very similar chemical structures [7]. The information on this training and validation dataset is provided here for context, and the performance of the “new model” on this dataset for DTI has been extensively reported in the previous study [7].

The primary application dataset consisted of the adverse effect-related targets aggregated from the Bassan et al., 2021 [9] and Smit et al., 2020 [8] studies (Table 1B). The targets were linked to their adverse effects specified in these studies and to their corresponding protein sequences from the UniProt database. Since targets are already categorised by organ groups in the Bassan et al., 2021 [9] study, the targets were also grouped here into organ-related categories for the Smit et al., 2020 [8] study, specifying the main organs for which the adverse effects related to each target affect. Organ groups in the Bassan et al. 2021 [9] study were amended where appropriate to reflect the wider encompassing organs’ effects that may not have been previously included. Where the organs are wide encompassing, the category shall be assigned as ubiquitous.

The combined dataset was quality controlled by removal of duplicate targets (e.g., Endothelin ETB Receptor and Platelet-Activating Factor (PAF) Receptor), pooling together different organ-related information across duplicated targets, and incorporation of additional adverse effects information from the literature. Additional organ sources were added where appropriate. For example, Skin was added in addition to Lung/Heart for Histamine receptor H1, and Cerebral was added to Heart for 5-hydroxytryptamine receptor 1D. Additional adverse effects identified in the literature were also added to the existing list of adverse effects, where appropriate, e.g., Lung: idiopathic pulmonary fibrosis was added for Janus Kinase 2.

**Figure 1:**
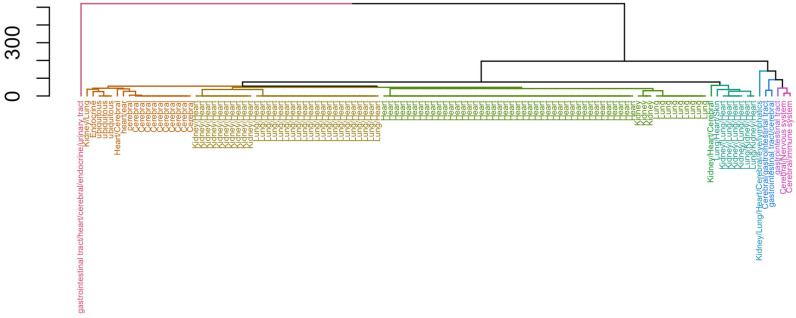
Clustering by the adverse effects of different organ sources using hierarchical clustering with Levenshtein distance.

A hierarchical clustering based on the adverse effects across different organ sources is shown in Figure 1. The dendrogram is cut at k = 10 to obtain 10 clusters in different colours. The organ sources are ranked in terms of ordering for a particular target, such that the organ source with a higher number of related adverse effects is ranked first. For example, for the ranking Lung/Heart/Kidney, the protein target would have more adverse effects related to the Lung than Heart, and Heart more so than Kidney.

The model evaluation was performed on the SIDER (Table 2) dataset as well as on the FAERS dataset for drugs whose adverse effects were not available in the SIDER dataset. A subset of the human proteome (*n* = 83,593 proteins) from the UniProt database was extracted and used as the negative control dataset. This was performed with the assumption that the human proteins, excluding the adverse effect-related target proteins in the main dataset, are not associated with adverse effects. The human proteome control dataset was filtered to remove the adverse effects dataset-related proteins (*n* = 118 unique proteins), including any isoforms. Due to high computational cost (i.e., it takes approx. 24 h to compute using the entire human proteome per compound), 1000 proteins were randomly sampled from the entire human proteome for use as this control dataset.

The selection of clinical compounds and targets was based on (i) the existing guidelines: the British National Formulary (BNF); (ii) commonly used compounds in the United Kingdom healthcare system; (iii) clinical expertise/prioritisation from the clinical pharmacist, cardiologist, and cardiac surgeon; (iv) previous review work conducted, focusing on relevance for the treatment and management of AF [10].

### 2.2. Target Featurisation

The chemical compounds were transformed into features using the Morgan fingerprint method. The protein sequences were transformed into features using the Skipgram Neural Network embedding approach, as described by ProtVec [11]. The rationale for using this embedding approach over that of ProtBERT, as in [12], is that the latter requires CUDA (NVIDIA GPU) to be available and configured to the appropriate version of the torch library on the device, which is not always available, as is the case in this study. In addition, ProtBERT requires enormous computational power and continuous, rapid connectivity to platforms such as Hugging Face, which may also pose challenges.

### 2.3. Modelling Approach

Based on a priori test set prediction performances of PR AUC 0.813, a deep cross-modal attention model was applied for the evaluation of the adverse effects dataset. The model evaluation of the adverse effects dataset used the contrastive learning “new model” from a previous study [7] as it was expected that the regularisation-like effect of contrastive learning would be helpful in terms of generalisation. This modelling approach is summarised below.

The embeddings $E_{i}$ = $f_{i}\left(X_{i}\right)$ of the target (*i* = 1) and compound (*i* = 2) are entered separately into 3-layer feed-forward projection modules:

(1) $$\begin{array}{c} L_{1}^{i}=ReLU(W_{1}^{(i)T}E_{i}+b_{1}^{i})\\ L_{2}^{i}=ReLU(W_{2}^{(i)T}L_{1}+b_{2}^{i})\\ L_{3}^{i}=ReLU(W_{3}^{(i)T}L_{2}+b_{3}^{i}) \end{array}$$

where *W* represents the weights and *b* represents the bias in each layer. Subsequently, a dual-headed cross-modal self-attention component is added:

(2) $$A=\;\left[\sigma \left(\frac{Q^{1}K^{2}}{\surd (p/2)}\right)V^{2},\;\sigma \left(\frac{Q^{2}K^{1}}{\surd (p/2)}\right)V^{1}\;\right]W_{G}$$

where p=1024, $W_{G}$ is the weight capturing the global contextual information across attention heads, $Q^{1}$ is the query matrix calculated using the target embedding, and $K^{2}$ and $V^{2}$ are calculated using the drug compound embeddings in the first attention head. In the second head, this is reversed, where $Q^{2}$ is the query matrix calculated using the drug compound embedding, and $K^{1}$ and $V^{1}$ are calculated using the target embeddings. This composition is used to maximise both the local and global information captured across the two modalities.

Subsequently, residuals were added, and layer normalisation was performed to propagate the gradient and normalise features. Finally, a single-layer feed-forward linear classifier is added to conduct the classification. Binary cross-entropy (BCE) loss with the sigmoid function was used for the optimisation process rather than standard BCE loss to ensure values are normalised strictly between 0 and 1 during this process.

(3) $$W_{4}^{T}\left(\frac{\hat{A}-\mu_{\hat{A}}}{\sqrt{\sigma_{\hat{A}}^{2}+\varepsilon }}\;\alpha +\beta \right)+b_{4}$$

where $\hat{A}=E_{1}+\;E_{2}+A,\;{\;\mu }_{\hat{A}}$ is the empirical average of $\hat{A}$, $\sigma_{\hat{A}}^{2}$ is the variance of $\hat{A}$, and α and $W_{4}^{T}$ are the weights of the normalisation and classification layers, respectively. β and $b_{4}$ are the bias of the normalisation and classification layers, respectively. ε is a small constant in the denominator that reduces the likelihood of numerical instability.

The above modelling process was applied to the BIOSNAP dataset as initial pre-training [7]. This was then followed by contrastive learning as per [12], using the DUD-E training dataset. The Triplet (contrastive) loss function was used to perform the contrastive learning component of the training process. For each drug compound interaction pair, 50 non-interacting decoys were sampled randomly to produce the triplets, and the loss values were averaged across each triplet set. To explain in more detail, the DUD-E dataset of positive samples with random samples of negative sample combinations used to facilitate the contrastive learning were only applied to the MINER-DTI [7] training dataset, such that it aimed to enhance the contrast between the MINER-DTI training dataset’s positive and negative sample pairs for drug–target interactions whilst maximising the likelihood of interaction between the active compound in the DUD-E dataset and the target in the MINER-DTI training dataset. In this sense, the DUD-E’s decoy non-active samples act like an augmentation to the negative samples in the MINER-DTI training dataset.

Further details on the modelling approach are provided in the Supplementary Materials, Modelling Details section. Details on computational resources are provided in Supplementary Materials, Table S1.

### 2.4. Representation Approach

Due to projection to the latent space resulting in negative embedding values, which can have arbitrary spatial meanings in terms of its negativity (i.e., the negative spatial orientation is not an indication of dissimilarity by itself), the cosine similarity was not used, but Euclidean distance was used instead, which cannot be negative. Due to the curse of dimensionality, Euclidean distances may not be robust in directly calculating the distance in non-linear distances across high-dimensional data functions. Hence, the PaCMAP approach was applied to preserve the global and local structure of data in a lower-dimensional space (i.e., two-dimensional projection space) before calculating the Euclidean distance [13]. This distance metric is then a measure of the predicted extent of interaction between targets and compounds, with smaller distances representing higher interactions. The model embeddings of the adverse effect-related targets, human control proteins, and anticoagulant compounds were projected, and distances were calculated using this approach.

### 2.5. Text Normalisation

Text for drug adverse effect terms is often non-standard, creative, or colloquial, and discrepancies can exist across the SIDER/FAERER ground truth and model predictions, even for semantically equivalent terms. Hence, text normalisation was applied by mapping the ground truth set and prediction set of adverse effect terms to the MedDRA (Medical Dictionary for Regulatory Activities) Preferred Terms (PTs) [14]. For text normalisation, the UKNLP (University of Kentucky) Hierarchical RNN with LSTM was previously reported to perform highly with an accuracy of 87.2% and hence was applied for normalising the ground truth and prediction terms [14].

### 2.6. Model Evaluation

The new model was benchmarked against the contrastive model in [12], whereby the only difference is that the ProtBERT featurisation of targets is replaced with using Skipgram Neural Network embedding instead. As the process is still very computationally costly and the focus of this study is on external evaluation to a different task domain (adverse effects instead of drug–target interaction) [7], the evaluation methodology is applied to the test dataset instead of the internal validation set [15]. Precision and recall metrics were used to evaluate the performance of models rather than PR AUC, since for this task domain, the predictions are normalised adverse effect terms rather than probabilities of interaction.

## 3. Results

The evaluation of model performances shows that the SIDER dataset compounds showed a trend towards higher recall than precision (Table 3), with rivaroxaban showing the highest recall (0.746), followed by dabigatran (0.702), and all models showing a higher precision and recall compared to the ConPlex model, which failed to attain satisfactory prediction in any of the tasks.

The FAERS dataset compounds showed substantially higher precision compared to the SIDER dataset compounds, with enoxaparin achieving the highest precision (0.879) and edoxaban obtaining the second highest precision (0.770).

**Figure 2:**
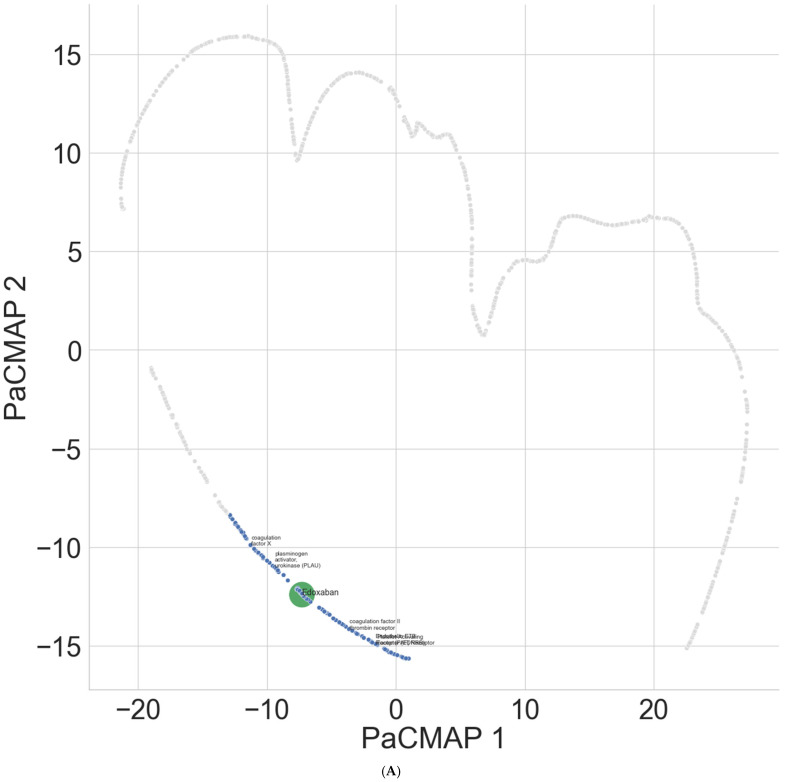

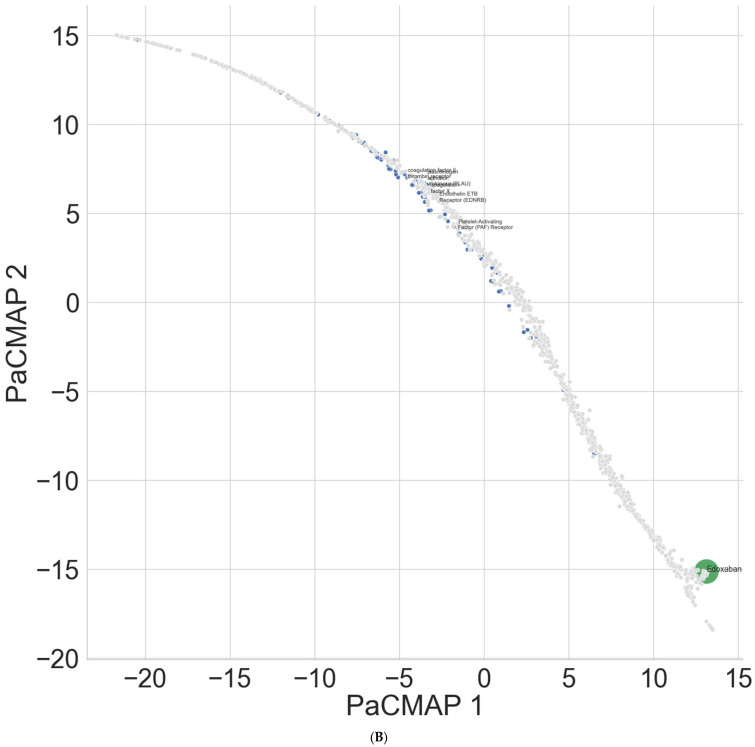
Using PaCMAP, the learned latent space for Edoxaban (green), adverse effect-related targets (blue), and human protein controls (grey) are shown for (**A**) new model and (**B**) ConPlex.

Edoxaban was shown to be closely interacting with the set of adverse effect-related proteins in the new model (Figure 2A). The control proteins were shown to interact at a further distance to edoxaban compared to the adverse effect-related proteins, suggesting a reduced likelihood of interaction and off-target effects with edoxaban. The ConPlex model showed implausible interactions and that the interactions between edoxaban were closer to the human control proteins compared to the adverse effect-related proteins, further explaining the failure to attain satisfactory prediction in terms of precision and recall (Figure 2B).

**Figure 3:**
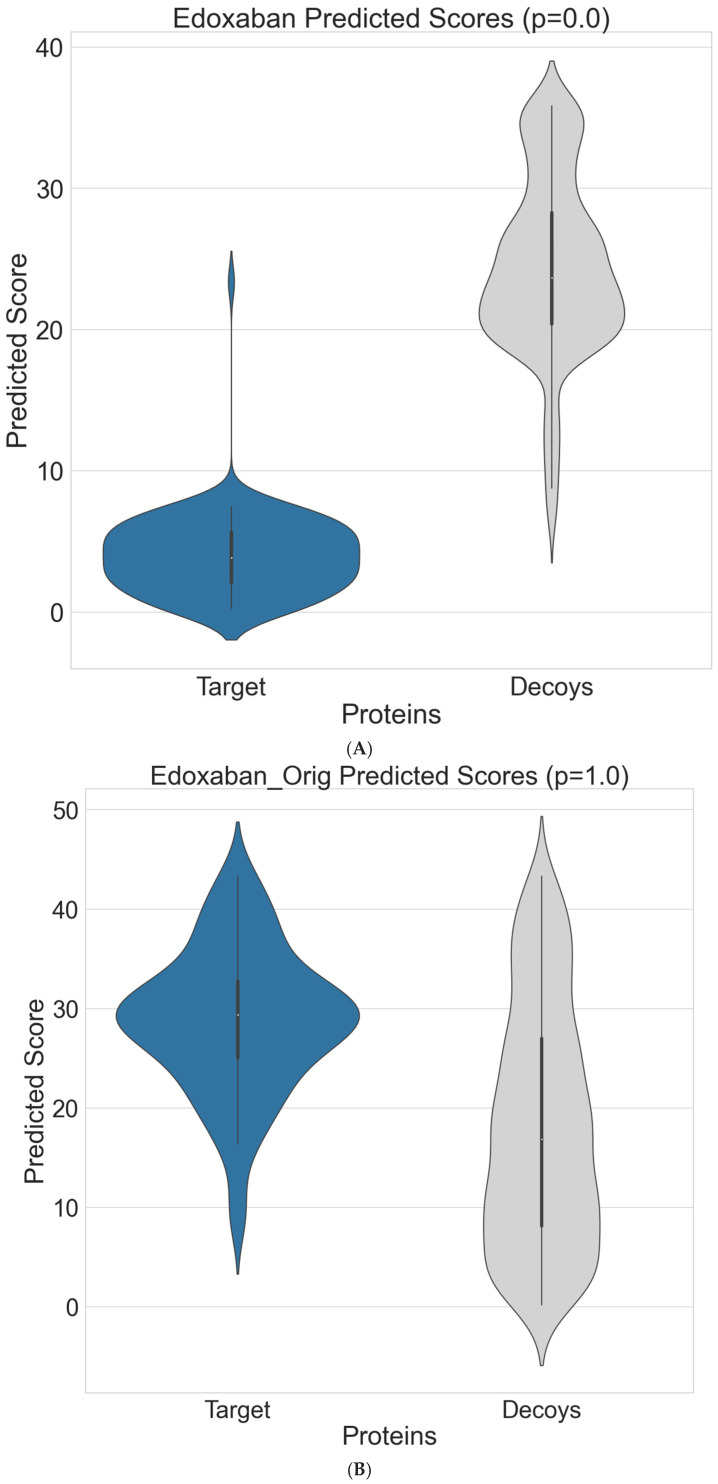
Using a violin plot, the distributions of interaction scores for edoxaban against the adverse effect-related targets (blue) and human protein controls (grey) are shown for (**A**) new model and (**B**): ConPlex; *p*-value shows results from a one-sided *t*-test.

These results are confirmed in Figure 3A, which shows that the new model found that edoxaban interacted more strongly with the adverse effect-related targets compared to the human protein controls (*p* < 0.001), whereas the ConPlex model failed to identify any differences across the distribution of interaction scores in the two groups (*p* = 1.0; Figure 3B).

For the compounds in the SIDER dataset, apixaban was found to have the optimal safety profile in terms of the strongest interaction with the target of interest (Ranking 47; Table 4), coagulation factor X, whilst having the least off-target effects in terms of bleeding-related adverse effects (see values in bold). It can be seen that dabigatran and warfarin also strongly interact with the factor II thrombin cascade. Compared to rivaroxaban, warfarin can be seen to have mildly stronger off-target interactions with other bleeding adverse effect markers, except for PLAU, which showed weak interactions.

For the compounds in the FAERS dataset, it can be seen that enoxaparin shows the best efficacy profile in terms of bleeding, showing the strongest interaction with coagulation factor X (Ranking 34; Table 5), whilst having a strong safety profile in terms of low off-target effects on other bleeding-related markers (see values in bold). The natural compound sequoiaflavone can be seen to have a similar pattern of interaction ranking compared to rivaroxaban, suggesting potential similarities in action. The strong binding of edoxaban to multiple off-target bleeding-related adverse markers suggest that it would have stronger bleeding related adverse effects than other compounds such as apixaban and enoxaparin. However, it can be seen that edoxaban has stronger interaction specificity for coagulation factor X than rivaroxaban (Ranking 66 vs. 215), with lower off-target interaction for all other bleeding markers except plasminogen activator urokinase (PLAU).

**Figure 4:**
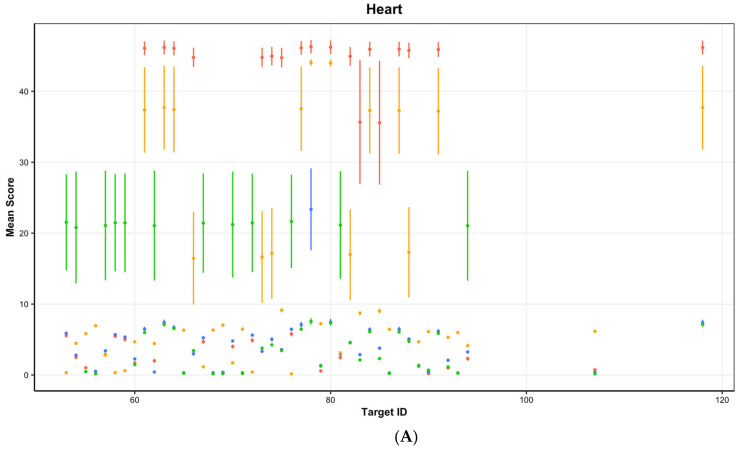

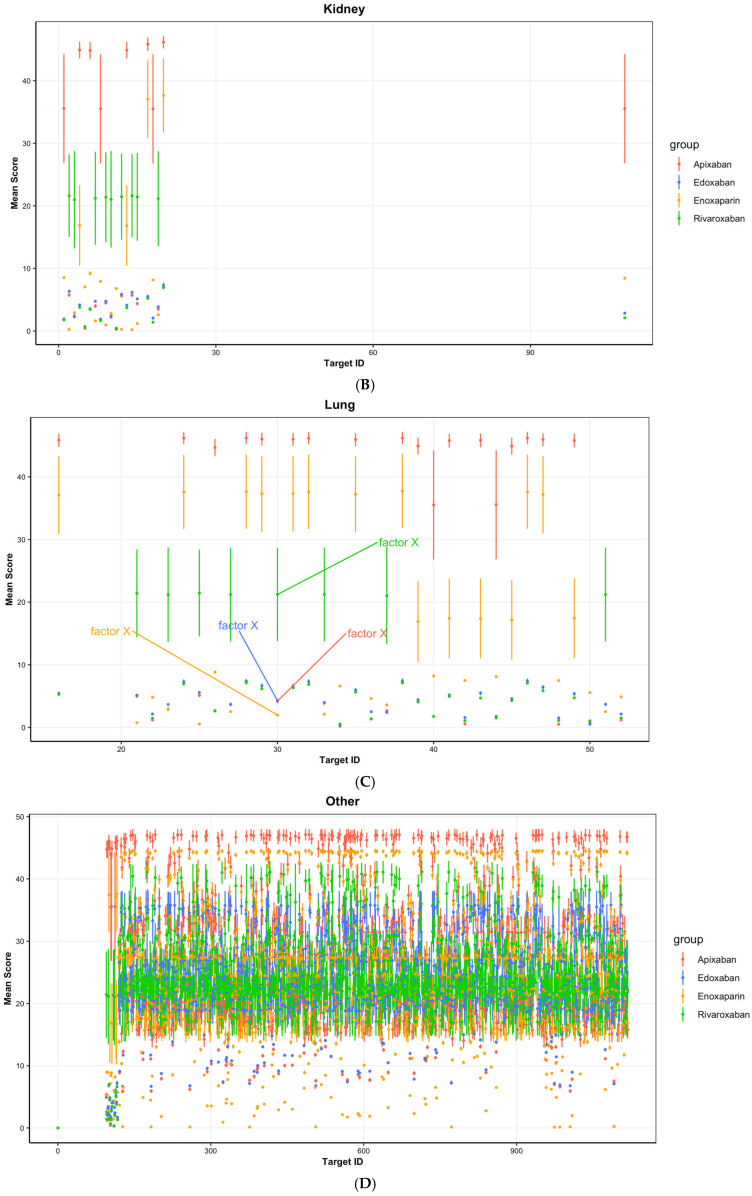
Plot shows all adverse effect-related proteins simultaneously comparing Euclidean distance scores to the four compounds, edoxaban, rivaroxaban, enoxaparin, and apixaban across (**A**): Heart; (**B**): Kidney; (**C**): Lung; (**D**): Other organ types; point and error bars show the mean and standard error of Euclidean distance scores across five repetitions; red—apixaban; blue—edoxaban; orange—enoxaparin; green—rivaroxaban.

In terms of overall adverse effects extending beyond only bleeding-related ones, more adverse effects are shown to affect the heart, lung, and other organs than the kidney (Figure 4). In terms of the heart, lung, and kidney, apixaban and enoxaparin showed the lowest level of off-target adverse effect-related interactions, with rivaroxaban and edoxaban showing similar levels of strong interacting adverse effects. A similar pattern is observed in the other organ group. However, a higher proportion of strong adverse effect marker interactions, with interaction Euclidean distance scores closer to 0, are observed in the other organ group for enoxaparin compared to edoxaban.

Sequoiaflavone was confirmed to have a similar profile to rivaroxaban, with substantially fewer adverse related targets having very strong interactions in the other organ group compared to enoxaparin (Supplementary Materials, Figure S1).

## 4. Discussion

Previous applications of MINER-DTI and related models have primarily focused on the task of drug–target interaction prediction without considering adverse effects prediction or text normalisation. Here, a deep cross-modal attention pipeline was extended, applied, and evaluated to a different task domain of adverse effects screening, with text normalisation modelling to support performance evaluation. Since each target can be associated with many different adverse effects, and text for drug adverse effect terms are often non-standard even for semantically equivalent terms, the text normalisation was essential to enable domain differences across datasets to be synchronised to the same set of MedDRA PTs.

Whilst text normalisation models have been applied for adverse effects’ term normalisation before [14], these have generally not been considered in the context of drug target screening for the MINER-DTI dataset. In addition, while previous models that were designed specifically for DTI such as ConPlex failed to perform in the new task domain of adverse effect screening [12], the new model showed prediction potential for anticoagulant screening, especially for the FAERS anticoagulant compounds. One possible reason for this is that the compounds within this dataset had a larger more complete set of reported adverse effects than SIDER due to longitudinal monitoring over an extended number of years in the United States [16].

Whilst the combined application dataset was quite small (*n* = 118), the model was trained on a much larger MINER drug–target interaction (DTI) dataset with 2181 targets. In addition, contrastive domain adaptation (a form of transfer learning) using a triplet loss function with data augmentation from the DUD-E dataset was applied to minimise the risk of overfitting and generalizability issues when applied to the external dataset.

Adverse effects primarily occur due to the lack of adequately specific molecules acting on the intended targets. Currently, the arsenal of molecules available to hospitals may not be specific enough. That is, off-target effects can lead to more side effects than desirable. This not only increases the likelihood of failed clinical trials but also the likelihood of drug non-persistence in approved drugs. The major challenge is that one typically cannot know the likelihood of significant adverse effects until after clinical trials have been performed, whether in vitro or in vivo. By then, if the drug fails, as exemplified by asundexian, enormous resources (including budget, time, effort, and animal or patient suffering) are likely to have been spent.

One of the strongest arguments in favour of using advanced technology is the possibility of screening for off-target effects earlier on, as demonstrated in the present study. That is, if protein mapping against the drug adverse effects could be performed, this could improve the specificity of drug effects, potentially guiding future efforts to minimise off-target effects by further refinements to the drug or through the identification of better alternatives.

The existing molecular docking methods are limited in that they cannot calculate affinities accurately nor even reliably rank order high-scoring molecules, although hit rates are often beyond 10% [17]. In addition, these methods require manual modifications to proposed potential areas of binding, and drug targets need to be laboriously processed individually, one at a time. Nonetheless, these remain useful for explaining molecular interactions for newly identified molecular interactions.

Deep learning-based drug interaction screening approaches have the potential for high precision-recall performance and can offer substantially faster screening of many potential target or compound candidates [12]. However, it should be noted that the Euclidean distance in a latent space is a useful proxy but it requires rigorous experimental validation and further clinical trials to confirm its correlation with real-world toxicity. As such, the focus of this study is on a screening and prioritisation tool, not a definitive predictor of clinical outcomes. Through further improvements in modelling approaches, the reliability of in silico methods will improve over time. Eventually, this could lead to a step-change in the way animal and clinical trials are conducted, as well as the way decisions on drug prescription are made. That is, improvements in in silico techniques could result in increased confidence that the drug will be effective and successful in clinical trials. Additionally, improved modelling will aim to reduce adverse effects by being more effective in filtering out high-risk drugs faster. It will also aim to allow optimised drugs (considering both risk and benefit trade-offs) to pass through trials more successfully.

Numerous studies have compared the actions of apixaban on activated coagulation factor X to those of rivaroxaban. The current study concurs with previous studies’ findings that apixaban is the most potent inhibitor of factor X, with an inhibitory constant (Ki) of 0.08 nM compared with a Ki of 0.4 nM for rivaroxaban [18]. However, pharmacokinetic profiles of rivaroxaban are more favourable, binding to factor X with a 4-fold faster association rate and 1.5-fold lower dissociation rate than apixaban [19]. Interestingly, disparities in the clinical application of these two drugs are also present, whereby apixaban followed by rivaroxaban are the most commonly used drugs after warfarin in human patients [20], whilst the use of rivaroxaban is more widely reported in veterinary patients. However, few studies have analysed their relative potential for adverse effects across a wide range of known targets associated with adverse effects.

Here, it was found that rivaroxaban and warfarin showed a similar degree of off-target interactions for bleeding markers, with warfarin having mildly stronger interactions except for plasminogen activator urokinase, a thrombolytic agent that is associated with risk of haemorrhage [21]. This is consistent with evidence that these two anticoagulants have similar risks for bleeding, with warfarin having a lower risk of gastrointestinal bleeding and a higher risk of intracranial haemorrhage compared to rivaroxaban [22].

This study found that apixaban and enoxaparin showed strong efficacy in terms of high interaction with the desired target factor X, whilst having low bleeding adverse effects related to off-target interaction. This is consistent with evidence that suggested that edoxaban is generally associated with more bleeding events than enoxaparin [23], although the incidence of overall adverse events, including non-bleeding events, tends to be higher in the enoxaparin group than the edoxaban group [23].

This study also found that strong binding of edoxaban to multiple off-target bleeding-related adverse markers suggests that it would have stronger bleeding-related adverse effects than other compounds, such as apixaban. This is supported by evidence showing that edoxaban is associated with a higher risk of major bleeding, especially gastrointestinal bleeding, compared to apixaban [24]. In addition, we found that edoxaban has stronger interaction specificity for coagulation factor X than rivaroxaban, with lower off-target interaction for all other bleeding markers except plasminogen activator urokinase (PLAU). This is supported by existing research, which has shown that edoxaban is associated with a lower risk of bleeding compared to rivaroxaban [25].

Due to the specific targeted designs of apixaban for human activated coagulation factor X, it is expected that it would have fewer off-target effects compared to sequoiaflavone. However, here it was found that the factor X binding and safety profiles of sequoiaflavone were very similar to those of rivaroxaban. These results provide additional means for biologists and pharmacology researchers to investigate the ligand optimisation of sequoiaflavone to increase its target selectivity and binding affinity in order to further assess its optimised potential for minimising adverse effects, whilst maintaining activity.

Whilst pharmacists commonly use the electronic medicines compendium in terms of identifying adverse effects when making risk vs. benefit analyses in drug-related decision making, the majority of the adverse effects for apixaban identified there were bleeding/haemorrhage-related adverse effects. However, the biological targets related to such effects are not provided. In addition, the adverse effects provided in the EMC did not include many of the commonly reported adverse effects listed on the NHS patient leaflets or on other medication websites.

The disconnection between the information provided by different sources means that clinical pharmacists are not provided with such prior information, which influences drug non-persistence. This can limit the effectiveness of risk vs. benefit analysis. It also suggests the need for approaches similar to those presented here, whereby siloed sources of information are connected through the screening of adverse effect-related targets.

Currently, bleeding-related adverse effects can be monitored through testing the blood for unbound levels of factor Xa level (as is performed for warfarin, which is decreasingly used). However, there is currently no clinical monitoring recommended in relation to apixaban for bleeding-related adverse effects in humans and animals. This may be associated with the relatively low level of adverse effects related to its off-target interactions.

In addition to preventing thrombosis, existing factor X drugs inhibit the process of fibrin formation, which is essential to haemostasis, hence increasing the risk of bleeding [26]. Factor XI drugs such as abelacimab are currently undergoing phase III trials [5]. Such factor XI inhibitors are likely to offer reduced adverse effects than their factor X counterparts due to the ability to uncouple thrombosis from haemostasis [26]. This is therefore currently the focus of much research in the area of anticoagulation [27]. One challenge is that before all four phases of drug clinical trials are complete, the pharmaceutical companies will not release the chemical structure details, making pre-emptive adverse effect modelling more challenging and largely feasible only within pharmaceutical companies.

Nonetheless, the approaches developed here will also be useful for assessing likely risk vs. benefit for novel compounds that have recently completed clinical trials. This is especially the case for hospital management and clinicians who have to decide on the usage of multiple different drug candidates. In doing so, they could be aided with the simultaneous consideration of the biological profile of the patients, which might contraindicate the use of certain drugs. In addition, the assessment of pre-clinical trial natural compounds, such as those considered herein, can also fuel further future efforts in adding to the repertoire of drugs as part of the drug discovery process.

One interesting consideration is the clinical differences that approaches like the one in this study would make. That is, if one has such knowledge of target interaction ranking and associated adverse effects in advance, would it influence the decision making in terms of what drugs are prescribed clinically? The important strategy to consider here is the impact the approaches will have on the risk vs. benefit analysis, which is typically what pharmacists and clinicians consider when making drug prescription decisions, that is, the likely benefit the compound will have on the patient, against the potential risk for adverse effects. With additional developments in this area, it is envisaged that more confidence will be gained in providing the increasingly detailed information required for these professionals to come to more optimal risk vs. benefit decisions.

## 5. Future Work and Limitations

One general challenge exists in terms of determining if a patient’s disease became worse due to an adverse effect of a drug or due to progression of their underlying disease condition. Future work may aim to disentangle the effects of ageing and disease progression from the adverse effects of drugs. In addition, newly identified targets related to adverse effects can provide the means for further validation by biologists, pharmacologists, and clinicians through in vitro and in vivo trials. However, the model’s predictions are limited to interactions with the known targets and cannot yet predict truly novel, previously unassociated adverse effects. New experimental discoveries of relationships across previously unassociated targets and their linked adverse effects may therefore be one useful means for improving the screening tool by updating both the dataset and model considered.

As the proteins and adverse effects considered here relate to only those in Homo Sapiens, future studies may also investigate the level of interactions in protein targets of animals such as cats and dogs for rivaroxaban, apixaban, and sequoiaflavone. Although there has been a phase I trial of apixaban in dogs [28], the side effects of apixaban in cats are largely unknown since currently there is not sufficient evidence on its use for it to be licenced in this species [29]. The limited studies to date have pertained to pharmacokinetics and pharmacodynamics [30]. Since many proteins are quite conserved across mammalian species, one would expect similar effects in dogs and cats. This would provide impetus for future studies to examine a specific protein set for cats in terms of interaction with apixaban.

Clinical trials generally start with the trial in earlier phases on animals before progressing to humans, should the former show success. One limitation of translating adverse effects between animals and humans is that the sets of adverse effects commonly observed in animals and humans may differ. It can be challenging to identify more subtle side effects in patients unable to report symptoms (e.g., headaches). Nonetheless, other methods of detecting adverse effects, such as diarrhoea, monitoring of urine output, and echocardiographic determination of left atrial dilatation as surrogate markers of adverse effects, can be considered in those scenarios.

This study focused on the adverse effects of anticoagulation compounds that have potential for managing atrial fibrillation. Future studies should also consider antiarrhythmic compounds and natural alternatives in relation to their adverse effects. For example, amiodarone has a large range (more so than other antiarrhythmics) of very severe adverse effects, including arrhythmias, hepatic disorders, hyperthyroidism, nausea, pulmonary fibrosis, delirium, angioedema, nerve disorders, and severe cutaneous adverse reactions, among others [31]. Bisoprolol has fewer adverse effects in comparison. However, in clinical practice, clinicians will use a risk–benefit analysis to select the most appropriate drug for an individual patient [32].

Factor XI drugs are further up in the clotting factor-related cascade than factor X drugs. They may hence pose a future for anticoagulation therapy with potentially fewer off-target related side effects [33]. As the chemical structure of abelacimab was not available, future efforts shall aim to further research this compound when the four phases of clinical trial are complete.

Future work should also aim to use approaches such as computer-aided synthetic planning (CASP), structure-based virtual screening (SBVS), and pharmacophore-based ligand-based virtual screening (LBVS) to design drugs that are as specific as possible. This will aim to minimise off-target systemic effects and improve both selectivity and specificity [34]. It may, to a small extent, involve the optimisation of drug synthesis routes through deep learning [35] but may also rely on generative AI-based methodologies [34].

Clinical trial failure results mainly from toxicity and suboptimal efficacy. Future studies should therefore aim to simultaneously quantify both adverse effects and treatment efficacy in terms of risk vs. benefit analysis [34]. Efficacy prediction, for example, can be examined from the perspective of linking transcriptomic data to clinical outcomes for prediction using graph-based approaches such as In silico Pathway Activation Network Decomposition Analysis [36,37].

## 6. Conclusions

Existing clinical pathways for the treatment and management of atrial fibrillation are limited by having to face the problems of adverse effects that occurred before assessing clinical risk vs. benefit decisions in terms of drug modifications. This study presents a framework that could be used to pre-emptively screen for adverse effects whilst considering the biological basis for guiding optimal drug selection before patients start medications and experience adverse effects. While the previous model, designed specifically for DTI such as ConPlex, failed to perform in the new task domain of adverse effect screening, the new model showed prediction potential for anticoagulant screening, paving the way for further development of the modelling approaches through future research efforts. One limitation is that compound dose and patient body/tissue-specific levels of protein targets were not available for incorporation into the model. This information, combined with patient-specific risk factors present in the compound-targeted population, could be beneficial for future improvements to this technique. In addition, future in vitro and in vivo studies may identify additional adverse effects previously unknown to be associated with the human proteome, thereby helping to extend the existing application dataset and model.

## Figures and Tables

**Table 1:** (**A**) Summary of the BIOSNAP and DUD-E dataset samples included. Training and validation dataset values represent the total number of combinations (number of interaction pairs/number of non-interaction pairs); the DUD-E dataset is only used in the training process, hence validation set boxes are left blank. (**B**) The combined dataset for protein target with associated adverse effects from two main studies; the counts show the number of adverse-related targets across organ groups for each of the two sub-datasets from Smit et al. 2020 [8] and Bassan et al. 2021 [9], respectively. In addition, a column showing the representative examples of adverse effects for each organ group is provided; ↑ increased levels; ↓ decreased levels; ↓/↑ disturbances.

(**A**)				
**Dataset**	**Drugs**	**Targets**	**Training**	**Validation**
**BIOSNAP**	4510	2181	19,238 (9670/9568)	2748 (1396/1352)
**DUD-E**	852,292	57	415,204 (8996/406,208)	—
(**B**)				
**Organ**	**Smit et al. 2020** [8]	**Bassan et al. 2021** [9]	**Representative Terms**	
**Cerebral**	11	0	drug abuse/dependence; drug abuse/dependence; heart rate increased; neurotoxicity; pain; acne; depression; dyskinesia; dystonia; parkinsonism	
**Cerebral/Nervous system**	1	0	dizziness; drug abuse/dependence; constipation; emesis; abdominal distension; apnoea; constipation; hard stool; hyperglycaemia; ischemic colitis	
**Cerebral/gastrointestinal tract**	1	0	inhibits pancreatic exocrine secretions; anxiogenic; causes vasoconstriction (venous); inhibits acid secretion; inhibits gastric emptying; inhibits gut motility; ↓ eating; ↓/↑ inflammation	
**Cerebral/immune system**	1	0	inflammation; lupus-like syndrome; sarcoidosis; ↑ pain; ↓ BP	
**Endocrine**	1	0	anaemia; ↓ attention; ↑ body weight; bone pain; depression; ↑ in hostility; hot flashes; impotence; ↓ memory; oedema	
**Heart**	1	41	angina, unspecified; angina, unstable; heart failure, abortion, alopecia, androgenic; alopecia, unspecified; heart failure; cardiomyopathy, ischaemic; cardiac hypertrophy; dermatological disease, unspecified;	
**Heart/Cerebral**	0	1	bipolar disorder; depression, major depressive, psychosis, unspecified; schizophrenia, disorder; headache, cluster; migraine;	
**Kidney**	0	3	arrhythmia; heart failure; death, cerebral complications, convulsions; ↑ urine excretion; death; oligoamnios; renal failure; hypocalvaria; limb defects; intrauterine growth retardation; persistent patent ductus arteriosus; respiratory distress syndrome; ↑ pain; ↑ urine excretion; ↑ urinary sodium excretion; haemolysis chronic dosing: neurodegeneration, ↑ urinary sodium excretion; heart: ↓ pain; ↓ HR; ↓ BP; AV block; cardiac	
**Kidney/Heart**	0	10	myocardial hypertrophy; perturbations of blood pressure, ↑ HR; ↑ thrombosis; hypertension, pulmonary; infarction, myocardial; nephropathy, diabetic; heart failure; ↓/↑ locomotor activity; drowsiness; ↓ urinary	
**Kidney/Heart/Cerebral**	0	1	fibrotic valvular heart, abuse/dependence; convulsions; ↑ arousal; psychosis; ↑/↓ locomotor activity; dyskinesia; perturbations of blood pressure; dizziness; ↑ urine excretion; neurodegeneration; headache;	
**Kidney/Lung**	0	1	renal dysfunction; gastric and pulmonary bleeding	
**Kidney/Lung/Heart**	0	4	alopecia, androgenic; cardiomyopathy, ischaemic; cognitive disorder, dermatological disease, unspecified; hypertension, unspecified; infarction, hyperuricemic, toxicity: ↑ risk spontaneous abortion; diabetic complication	
**Kidney/Lung/Heart/Cerebral/eye/lymphatics**	1	0	↑/↓ inflammation, ↓ uterine motility	
**Lung**	0	9	asthma; rhinitis; bipolar disorder; chronic obstructive pulmonary, bronchoconstriction, cardiac hypertrophy; Crohn’s disease; depression, unspecified; epileptic encephalopathy, foetal akinesia deformation, gastritis; incontinence, urinary; inflammation, hyperekplexia, hereditary	
**Lung/Heart**	0	21	acute coronary syndrome; angina, unstable; acidosis; irritability; ↓ pupil diameter; exhaustion; ptosis; muscle cramps; centrilobular liver, ↑ body temperature; sweating; atherosclerosis; infarction, cerebral; blood/clotting disease; cough; Buerger’s syndrome; hypertension, pulmonary;	
**Lung/Heart/Skin**	0	1	conjunctivitis, allergic; depression, major; eczema; eczema, atopic; insomnia; keratoconjunctivitis; ocular disorder; bipolar disorder; schizophrenia; skin disorder; urticaria; ↑ sleep; allergic, seasonal; depressive disorder; cardiac arrhythmia; ↓ GI transit;	
**Lung/Kidney/Heart**	0	2	↓ cardiac contractility; ↓ BP; bronchoconstriction; ↑ airway, excretion, hypertension; left ventricular, hypertrophy; bronchoconstriction; inflammation; pain; tubulointerstitial structural injury; ↓ HR; ↓ urine excretion; ↓ urinary sodium	
**gastrointestinal tract**	1	0	abdominal pain hypersensitivity; anxiety; bloating; cholelithiasis; cholestasis; fatigue; gas; nausea; pancreatic enzyme secretion; ↑ BP	
**gastrointestinal tract/cerebral**	1	0	inflammation; immunosuppression; ↑ GI transit; ↓ GI transit	
**gastrointestinal tract/heart/cerebral/endocrine/urinary tract**	1	0	facilitates gastrointestinal transit; diarrhoea; fainting; mechanical intestinal allodynia; ↑ GI transit; ↑ HR; ↑ QTc interval; ↓ BP; ↓ GI transit; ↓ blood volume	
**heart/ear**	1	0	atrial fibrillation; long QT syndrome; deafness; potential hearing impairment	
**ubiquitous**	3	0	insulin resistance; osteoporosis; convulsions; facilitates bronchial constriction; facilitates uterine constriction; facilitates vascular constriction; hypoglycaemia; immunosuppression; allergic inflammation; oedema	
**Total**	24	94		

**Table 2:** The adverse effects evaluation dataset and the corresponding anticoagulants; the number of adverse effects for each anticoagulant is presented.

	*N* Adverse Effects (SIDER)
**Dabigatran**	67
**Apixaban**	102
**Rivaroxaban**	89
**Warfarin**	56
	***N*** **adverse effects (FAERS)**
**Edoxaban**	612
**Enoxaparin**	1161

**Table 3:** Evaluation of the performance of adverse effect prediction models across compounds. **^a^** represents good prediction performance 0.70–0.80; **^b^** indicates excellent prediction performance 0.80–1.0.

	New Model		ConPlex	
	Precision	Recall	Precision	Recall
**Dabigatran**	0.314	**0.702 ^a^**	0	0
**Apixaban**	0.325	0.673	0	0
**Rivaroxaban**	0.366	**0.746 ^a^**	0	0
**Warfarin**	0.275	0.683	0	0
**Edoxaban**	**0.770 ^a^**	**0.703 ^a^**	0	0
**Enoxaparin**	**0.879 ^b^**	0.537	0	0

**Table 4:** Showing the interaction distance of SIDER compounds to bleeding-related markers; * represents the compound with the strongest binding to factor X; bold values show the compounds with the least off-target related interactions to other bleeding markers; the higher the ranking, the stronger the interaction, and vice versa for Euclidean distance.

	Dabigatran		Apixaban		Rivaroxaban		Warfarin	
	Euclidean Distance	Ranking	Euclidean Distance	Ranking	Euclidean Distance	Ranking	Euclidean Distance	Ranking
**Platelet-activating factor (PAF) receptor**	4.98	55	45.84	**984**	5.28	62	4.34	51
**Plasminogen activator urokinase (PLAU)**	9.13	90	2.63	37	21.04	199	25.19	**564**
**Endothelin ETB** **receptor (EDNRB)**	4.71	51	45.78	**979**	4.73	57	4.20	50
**Coagulation factor X**	9.69	101	4.20	47 *	21.22	215	25.95	631
**Coagulation factor II thrombin receptor**	3.54	40	44.74	**944**	3.45	45	2.37	33

**Table 5:** Shows the interaction distance of FAERS compounds (with Sequoiaflavone included as well for comparison) to bleeding-related markers; * represents the compound with the strongest binding to factor X; bold values show the compounds with the least off-target related interactions to other bleeding markers.

	Edoxaban		Enoxaparin		Sequoiaflavone	
	Euclidean Distance	Ranking	Euclidean Distance	Ranking	Euclidean Distance	Ranking
**Platelet-activating factor (PAF) receptor**	5.44	85	37.07	**895**	4.13	55
**Plasminogen activator urokinase (PLAU)**	2.40	37	3.60	57	18.26	**166**
**Endothelin ETB** **receptor (EDNRB)**	5.37	84	17.44	**336**	3.73	52
**Coagulation factor X**	4.28	66	1.98	34 *	19.37	186
**coagulation factor II thrombin receptor**	3.59	55	9.15	**143**	1.63	25

## Data Availability

The Bassan et al. dataset is available at: https://doi.org/10.1016/j.comtox.2021.100188 (accessed on 20 June 2025); The Smit et al. dataset is available at: https://doi.org/10.1021/acs.chemrestox.0c00294 (accessed on 20 June 2025); The sequence data is available at UniProt: https://www.uniprot.org/ (accessed on 20 June 2025).

## Supplementary Materials

The following supporting information can be downloaded at https://www.mdpi.com/article/10.3390/bioengineering12090972/s1, Figure S1: adverse effect-related proteins simultaneously comparing Euclidean distance scores to the four compounds, edoxaban, rivaroxaban, apixaban, with sequoiaflavone; Table S1: Evaluation of computational time and hardware cost performance of models; Modelling Details.

## References

[1] Piccini J.P., Caso V., Connolly S.J., Fox K.A.A., Oldgren J., Jones W.S., Gorog D.A., Durdil V., Viethen T., Neumann C. (2022). Safety of the Oral Factor XIa Inhibitor Asundexian Compared with Apixaban in Patients with Atrial Fibrillation (PACIFIC-AF): A Multicentre, Randomised, Double-Blind, Double-Dummy, Dose-Finding Phase 2 Study. Lancet.

[2] Asundexian Inferior to Apixaban for Stroke Prevention in Atrial Fibrillation. https://www.escardio.org/The-ESC/Press-Office/Press-releases/Asundexian-inferior-to-apixaban-for-stroke-prevention-in-atrial-fibrillation.

[3] García-Abeijon P., Costa C., Taracido M., Herdeiro M.T., Torre C., Figueiras A. (2023). Factors Associated with Underreporting of Adverse Drug Reactions by Health Care Professionals: A Systematic Review Update. Drug Saf..

[4] Verhamme P., Yi B.A., Segers A., Salter J., Bloomfield D., Büller H.R., Raskob G.E., Weitz J.I. (2021). Abelacimab for Prevention of Venous Thromboembolism. N. Engl. J. Med..

[5] Hardy J., Sperry A., Hartmann H., Goldfaden R., Ashchi M., Kim R., Huston J., Niman S., Choksi R. (2021). Abelacimab. Anti-Factor XI/XIa Monoclonal Antibody, Treatment of Atrial Fibrillation, Treatment of Thrombotic Disorders. Drugs Future.

[6] Apixaban 2.5 Mg Film-Coated Tablets-Summary of Product Characteristics (SmPC)-(Emc) 100159. https://www.medicines.org.uk/emc/product/100159/smpc.

[7] Dong T., Llewellyn R.D., Hezzell M., Angelini G.D. (2025). A Deep Learning Methodology for Screening New Natural Therapeutic Candidates for Pharmacological Cardioversion and Anticoagulation in the Treatment and Management of Atrial Fibrillation. Biomedicines.

[8] Smit I.A., Afzal A.M., Allen C.H.G., Svensson F., Hanser T., Bender A. (2021). Systematic Analysis of Protein Targets Associated with Adverse Events of Drugs from Clinical Trials and Postmarketing Reports. Chem. Res. Toxicol..

[9] Bassan A., Alves V.M., Amberg A., Anger L.T., Beilke L., Bender A., Bernal A., Cronin M.T.D., Hsieh J.-H., Johnson C. (2021). In Silico Approaches in Organ Toxicity Hazard Assessment: Current Status and Future Needs for Predicting Heart, Kidney and Lung Toxicities. Comput. Toxicol..

[10] Belfiori M., Lazzari L., Hezzell M., Angelini G.D., Dong T. (2025). Transcriptomics, Proteomics and Bioinformatics in Atrial Fibrillation: A Descriptive Review. Bioengineering.

[11] Asgari E., Mofrad M.R.K. (2015). Continuous Distributed Representation of Biological Sequences for Deep Proteomics and Genomics. PLoS ONE.

[12] Singh R., Sledzieski S., Bryson B., Cowen L., Berger B. (2023). Contrastive Learning in Protein Language Space Predicts Interactions between Drugs and Protein Targets. Proc. Natl. Acad. Sci. USA.

[13] Huang H., Wang Y., Rudin C., Browne E.P. (2022). Towards a Comprehensive Evaluation of Dimension Reduction Methods for Transcriptomic Data Visualization. Commun. Biol..

[14] Sarker A., Belousov M., Friedrichs J., Hakala K., Kiritchenko S., Mehryary F., Han S., Tran T., Rios A., Kavuluru R. (2018). Data and Systems for Medication-Related Text Classification and Concept Normalization from Twitter: Insights from the Social Media Mining for Health (SMM4H)-2017 Shared Task. J. Am. Med. Inform. Assoc..

[15] Gonzalez R., Nejat P., Saha A., Campbell C.J.V., Norgan A.P., Lokker C. (2023). Performance of Externally Validated Machine Learning Models Based on Histopathology Images for the Diagnosis, Classification, Prognosis, or Treatment Outcome Prediction in Female Breast Cancer: A Systematic Review. J. Pathol. Inform..

[16] DeLoughery E.P., Shatzel J.J. (2019). A Comparative Analysis of the Safety Profile of Direct Oral Anticoagulants Using the FDA Adverse Event Reporting System. Eur. J. Haematol..

[17] Lagos C.F., Segovia G.F., Nuñez-Navarro N., Faúndez M.A., Zacconi F.C. (2017). Novel FXa Inhibitor Identification through Integration of Ligand- and Structure-Based Approaches. Molecules.

[18] Ferri N., Colombo E., Tenconi M., Baldessin L., Corsini A. (2022). Drug-Drug Interactions of Direct Oral Anticoagulants (DOACs): From Pharmacological to Clinical Practice. Pharmaceutics.

[19] Kim P.Y., Yeh C.H., Dale B.J., Leslie B.A., Stafford A.R., Fredenburgh J.C., Hirsh J., Weitz J.I. (2018). Mechanistic Basis for the Differential Effects of Rivaroxaban and Apixaban on Global Tests of Coagulation. Open.

[20] Afzal S., Zaidi S.T.R., Merchant H.A., Babar Z.-U.-D., Hasan S.S. (2021). Prescribing Trends of Oral Anticoagulants in England over the Last Decade: A Focus on New and Old Drugs and Adverse Events Reporting. J. Thromb. Thrombolysis.

[21] Zeng J., Chen F., Chen Y., Peng M., Chen X., Yang Q., Wang R., Miao J. (2021). Predictors of Hemorrhagic Complications after Intravenous Thrombolysis in Acute Cerebral Infarction Patients: A Single-Center Study of 391 Cases. Medicine.

[22] Bai Y., Deng H., Shantsila A., Lip G.Y.H. (2017). Rivaroxaban Versus Dabigatran or Warfarin in Real-World Studies of Stroke Prevention in Atrial Fibrillation. Stroke.

[23] Fuji T., Fujita S., Kawai Y., Nakamura M., Kimura T., Fukuzawa M., Abe K., Tachibana S. (2015). Efficacy and Safety of Edoxaban versus Enoxaparin for the Prevention of Venous Thromboembolism Following Total Hip Arthroplasty: STARS J-V. Thromb. J..

[24] Chiv R., Beradid S., Suissa S., Renoux C. (2024). Effectiveness and Safety of Edoxaban Compared with Apixaban in Elderly Patients With Nonvalvular Atrial Fibrillation: A Real-World Population-Based Cohort Study. Stroke.

[25] Marston X.L., Wang R., Yeh Y.-C., Zimmermann L., Ye X., Gao X., Brüggenjürgen B., Unverdorben M. (2022). Comparison of Clinical Outcomes of Edoxaban versus Apixaban, Dabigatran, Rivaroxaban, and Vitamin K Antagonists in Patients with Atrial Fibrillation in Germany: A Real-World Cohort Study. Int. J. Cardiol..

[26] Fredenburgh J.C., Weitz J.I. (2023). News at XI: Moving beyond Factor Xa Inhibitors. J. Thromb. Haemost..

[27] (2024). Lipworth, K; Factor XI/XIa Inhibitors: What We Now Know. EMJ Cardiol..

[28] Gagnon A.L., Scansen B.A., Olver C., Shropshire S., Hess A., Orton E.C. (2021). Phase I Clinical Trial of an Antithrombotic Drug Protocol Combining Apixaban and Clopidogrel in Dogs. J. Vet. Cardiol..

[29] Welcome to the NOAH Compendium. https://www.noahcompendium.co.uk/home.

[30] Myers J.A., Wittenburg L.A., Olver C.S., Martinez C.M., Bright J.M. (2015). Pharmacokinetics and Pharmacodynamics of the Factor Xa Inhibitor Apixaban after Oral and Intravenous Administration to Cats. Am. J. Vet. Res..

[31] Amiodarone Hydrochloride Drugs BNF Content Published by NICE. https://bnf.nice.org.uk/drugs/amiodarone-hydrochloride/.

[32] Um K.J., McIntyre W.F., Healey J.S., Mendoza P.A., Koziarz A., Amit G., Chu V.A., Whitlock R.P., Belley-Côté E.P. (2019). Pre- and Post-Treatment with Amiodarone for Elective Electrical Cardioversion of Atrial Fibrillation: A Systematic Review and Meta-Analysis. EP Europace.

[33] Prakash S., Mares A.C., Porres-Aguilar M., Mukherjee D., Barnes G.D. (2023). Factor XI/XIa Inhibitors for the Prevention and Treatment of Venous and Arterial Thromboembolism: A Narrative Review. Vasc. Med..

[34] Fu C., Chen Q. (2025). The Future of Pharmaceuticals: Artificial Intelligence in Drug Discovery and Development. J. Pharm. Anal..

[35] Shi C., Gao T., Lyu W., Qiang B., Chen Y., Chen Q., Zhang L., Liu Z. (2024). Deep-Learning-Driven Discovery of SN3–1, a Potent NLRP3 Inhibitor with Therapeutic Potential for Inflammatory Diseases. J. Med. Chem..

[36] Swift L., Zhang C., Kovalchuk O., Boklan J., Trippett T., Narendran A. (2020). Dual Functionality of the Antimicrobial Agent Taurolidine Which Demonstrates Effective Anti-Tumor Properties in Pediatric Neuroblastoma. Investig. New Drugs.

[37] Ozerov I.V., Lezhnina K.V., Izumchenko E., Artemov A.V., Medintsev S., Vanhaelen Q., Aliper A., Vijg J., Osipov A.N., Labat I. (2016). In Silico Pathway Activation Network Decomposition Analysis (iPANDA) as a Method for Biomarker Development. Nat. Commun..

