# Supplementary material for: Rapid Screening of Anticoagulation Compounds for Biological Target-Associated Adverse Effects Using a Deep-Learning Framework in the Management of Atrial Fibrillation
Source: Bioengineering (Basel). 2025 Sep 12;12(9):972. doi: 10.3390/bioengineering12090972 (PMC12467381; doi:10.3390/bioengineering12090972)
Supplement: Supplementary file 1 [file bioengineering-12-00972-s001.zip › bioengineering-3835901-supplementary.pdf]

Supplementary Materials: Rapid screening of anticoagulation compounds for biological target-associated adverse effects using a deep-learning framework in the management of atrial fibrillation

Tim Dong, Rhys Llewellyn, Melanie Hezzell, Gianni D. Angelini

A

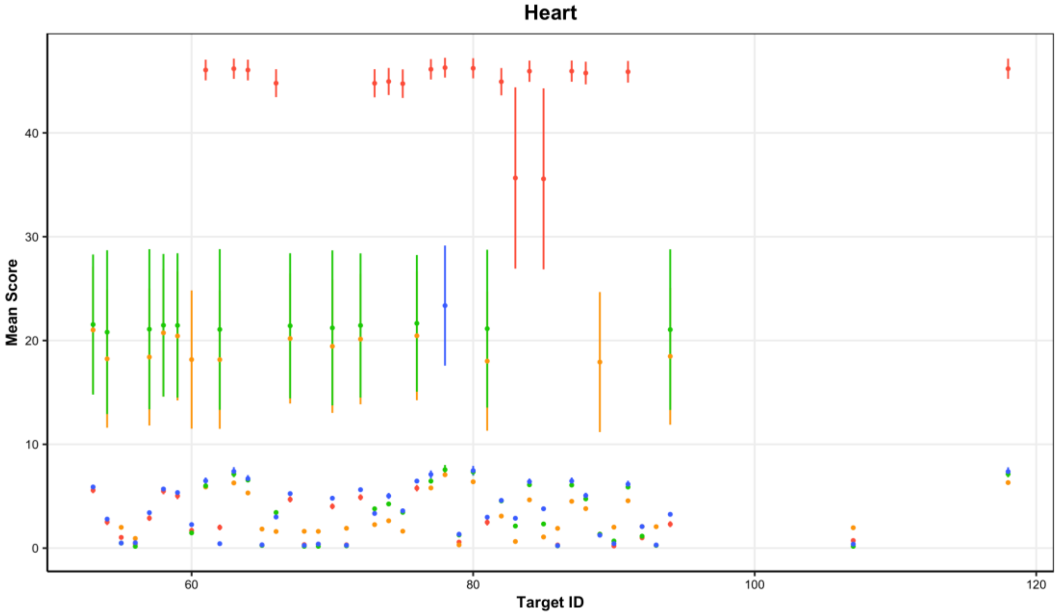

B

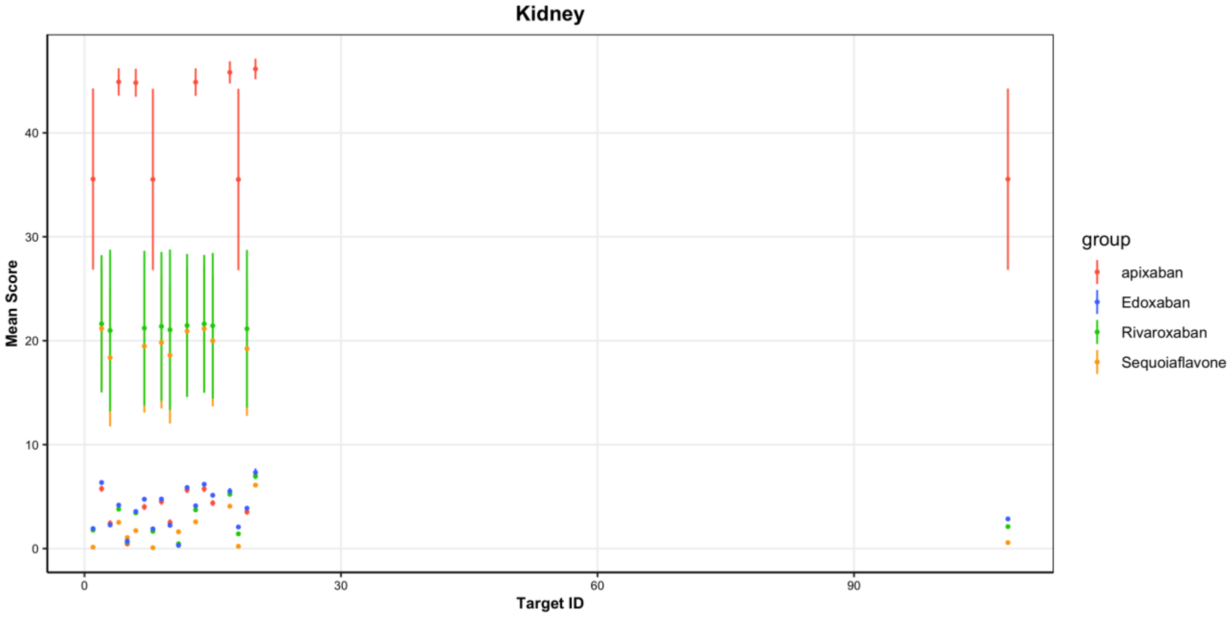

C

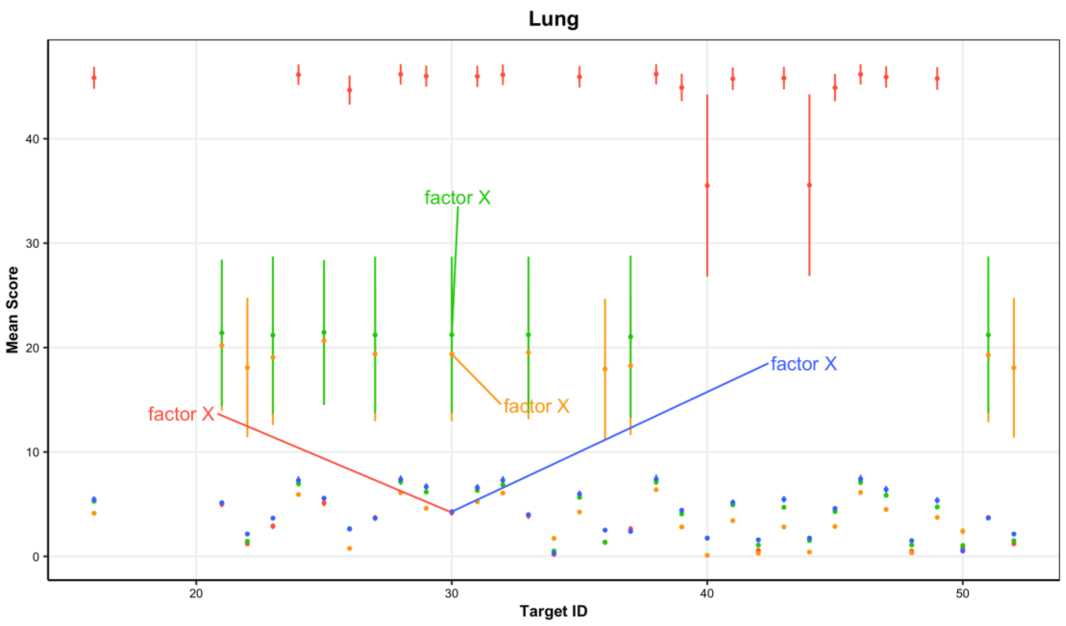

D

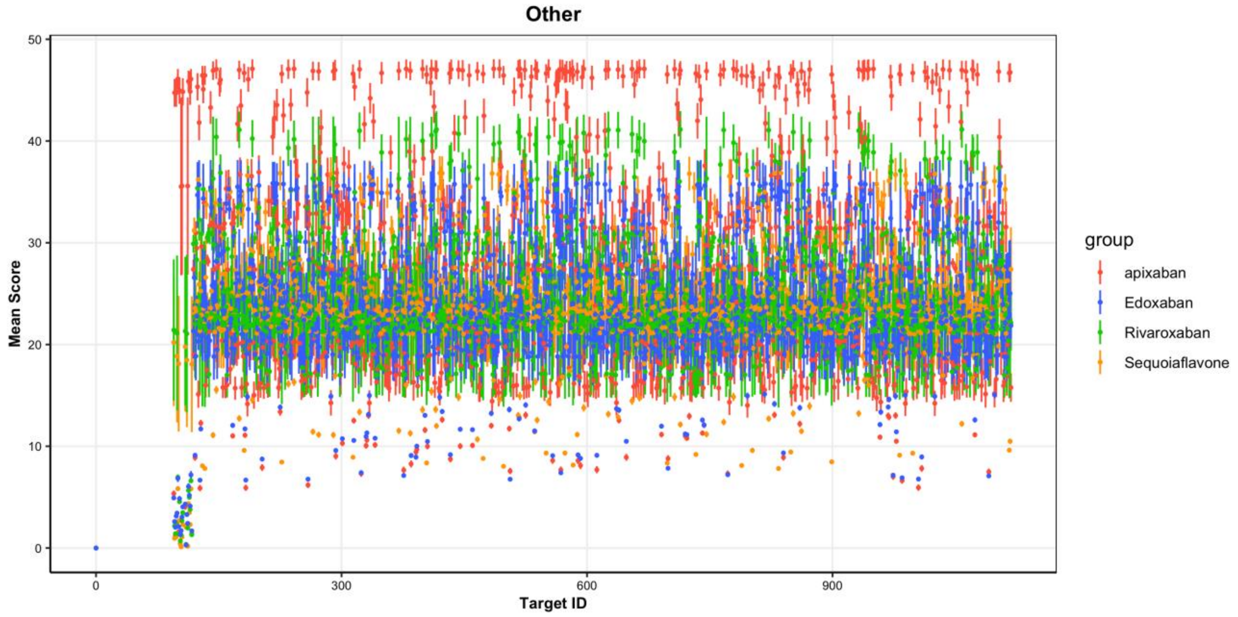

Figure S1. Plot shows all adverse effect-related proteins simultaneously comparing Euclidean distance scores to the four compounds, edoxaban, rivaroxaban, apixaban, with sequoiaflavone instead of enoxaparin across A: Heart; B: Kidney; C: Lung; D: Other organ types; point and error bars show the mean and standard error of Euclidean distance scores across five repetitions.

Modelling Details

The Xavier approach was used to initialise the model weights from a normal distribution.[1] The AdamW optimiser for error backpropagation was used to update the weights across a total of 50 epochs.[2] A cosine annealing schedule with warm restarts per 10 epochs was used to modify the learning rate,[3] which was initially set at 10^{-4}. The same annealing schedule approach was used for the contrastive problem, with the learning rate was initially set at 10^{-5}.[4] Using a tanh decay schedule with restarts every 10 epochs, the contrastive loss margin was first set at 0.25 and reduced to a minimum of 0 over 50 epochs. A batch size of 32 was used, along with an initial latent dimension of d_{α}=1,024 during drug and target featurisation and dimensions of d_H = 512 for each of the two attention heads.[5]

Table S1. Evaluation of computational time and hardware cost performance of models with contrastive learning (CL) in minutes against comparative ConPLex models here and that with GPU in original Singh et al [4] paper.

	Time (min) with CL	Hardware configuration	Hardware cost
**ConPLex**	69.4	14 inch MacBook Pro with Apple M1 Pro chip and 16 GB memory	Approx. £1,599.00
**New model**	165.8		
**Singh et al (ConPLex With GPU) [4]**	1273	112-core Intel Xeon Gold 6258R CPU and NVIDIA A100 GPU	Approx. £13,712 + £13,156.02 = £26,868

References

1. Glorot, X.; Bengio, Y. Understanding the Difficulty of Training Deep Feedforward Neural Networks. In Proceedings of the Proceedings of the Thirteenth International Conference on Artificial Intelligence and Statistics; JMLR Workshop and Conference Proceedings, March 31 2010; pp. 249–256.
2. Loshchilov, I.; Hutter, F. Decoupled Weight Decay Regularization 2019.
3. Loshchilov, I.; Hutter, F. SGDR: Stochastic Gradient Descent with Warm Restarts 2017.
4. Singh, R.; Sledzieski, S.; Bryson, B.; Cowen, L.; Berger, B. Contrastive Learning in Protein Language Space Predicts Interactions between Drugs and Protein Targets. *Proceedings of the National Academy of Sciences* **2023**, *120*, e2220778120, doi:10.1073/pnas.2220778120.
5. Dong, T.; Llewellyn, R.D.; Hezzell, M.; Angelini, G.D. A Deep Learning Methodology for Screening New Natural Therapeutic Candidates for Pharmacological Cardioversion and Anticoagulation in the Treatment and Management of Atrial Fibrillation. *Biomedicines* **2025**, *13*, 1323, doi:10.3390/biomedicines13061323.
